# How key family factors influence preschool children’s social–emotional competence: threshold effects of parental parenting styles and parent–child interaction quality

**DOI:** 10.3389/fpsyg.2026.1815141

**Published:** 2026-06-18

**Authors:** Yanru Wen, Cheng Zhu, Yiping Wei, Tianqing Chen, Tao Li, Xiaopeng Wu

**Affiliations:** 1 Faculty of Education, Guangxi Normal University, Guilin, China; 2 School of Education Sciences, Nanjing Normal University, Nanjing, China; 3 School of Education, Guangzhou University, Guangzhou, China

**Keywords:** parental parenting style, parent–child interaction quality, preschool children, social–emotional competence, threshold effect

## Abstract

**Introduction:**

Social–emotional competence is a core component of early childhood development. As the primary setting for its development, the family plays a crucial role, with parenting styles and parent–child interaction quality identified as key proximal factors. This study explores mediation and generalized summation models to examine the effects of these factors on preschool children’s social–emotional competence. It identifies specific score ranges within which authoritative and permissive parenting styles, respectively, promote or hinder children’s social–emotional competencies, thereby providing evidence-based reference thresholds for parental practices and offering empirical support for the development of early intervention programs tailored to the actual developmental needs of preschool children.

**Methods:**

A total of 504 preschool children aged 3–6 years and their parents in China were participated in a survey using the Social Skills Improvement System Social Emotional Learning Edition Rating Forms, the Parental Authority Questionnaire, and the Brigance Parent–Child Interactions Scale.

**Results:**

The findings revealed that: (1) Parental parenting styles and parent–child interaction quality are key familial factors influencing children’s social–emotional competencies, with the former exerting a primary effect; (2) Authoritative and permissive parenting styles predict children’s social–emotional competence positively and negatively, respectively. And parent–child interaction mediates the relationship between parental styles and children’s social–emotional competence; (3) In the generalized additive model incorporating parent–child interaction quality, authoritative and permissive parenting styles exhibit threshold effects on children’s social–emotional competence development: when authoritative parenting scores fall below 3.9, children’s social–emotional competence demonstrate a significant improvement across all domains. Conversely, when permissive parenting scores range between 2.5 and 3.7, children’s social–emotional competence experience a substantial decline.

**Discussion:**

This study reveals the nonlinear mechanism through which parental parenting styles and parent–child interactions jointly influence preschool children’s social–emotional competencies, providing a theoretical basis for optimizing family support strategies.

## 1 Introduction

Since the 1990s, the intensification of social issues impacting juveniles has prompted researchers to acknowledge the constraints of excessively “intellectualist” educational approaches. Consequently, there has been a paradigm shift toward recognizing the profound impact of non-cognitive abilities on student development, such as emotional intelligence. In recent years, as artificial intelligence and automation continue to reshape societal structures, social–emotional competence has emerged as a core capability for individuals to embrace themselves, coexist harmoniously with others, and sustain happiness in an ever-changing era. Its value has become increasingly prominent, making it a significant topic in the international fields of education and development. Social–emotional competence (SEC), originating from emotional intelligence theory, refers to the non-cognitive ability to recognize and regulate emotions, thereby guiding interactions with others and groups to achieve positive personal development (Salovey and Sluyter, 1997). The latest global assessment results released by the OECD (2023) indicate that children’s social–emotional competence holds unique value for navigating future societal complexity and variability, establishing it as a pivotal component in talent development (OECD, 2023). Specifically, this competency encompasses the manner in which individuals navigate relationships with themselves, others, and their communities (CASEL, 2020). It exerts a profound influence on academic adjustment, emotional understanding, interpersonal relationships, and social adaptation from early childhood onward (Domitrovich et al., 2017; Denham, 2018; Jones et al., 2019). Nevertheless, systematic research on the development of social–emotional competencies during the preschool stage remains relatively limited, and this domain has not received adequate attention in educational practice. This neglect may lead to young children exhibiting persistent social adjustment difficulties in real-world situations, experiencing difficulty integrating into peer groups, frequent aggression or withdrawal behavior during conflict; in addition to poor emotional regulation and vulnerability to strong and unstable emotional states (O’Connel et al., 2021). Consequently, this not only impedes the formation of healthy personalities and prosocial behaviors, but also significantly increases the risk of internalizing problems, including anxiety and depression, as well as externalizing issues, such as behavioral disorders (Greenberg et al., 2017). Therefore, it is of great practical significance to deeply explore the developmental mechanisms of social–emotional competence in preschool children.

The development of children’s social–emotional competencies unfolds as a continuum, shaped by the combined influence of multiple systemic factors including sociocultural contexts, interactive environments, peers, teachers, and families (Guralnick, 2010). Among the many influences, the family holds particular significance as the primary setting for socialization in preschool children, exerting a profound and lasting impact. Research indicates that family income and parental educational attainment are closely associated with children’s mental health (Christensen et al., 2017). Mothers with low levels of education, a history of psychological problems, or complex family structures are more likely to contribute to young children’s emotional and behavioral problems (Hancock et al., 2013; Schneider et al., 2017). The family environment has also been confirmed as a significant predictor of young children’s social–emotional competencies, with supportive environments exerting positive predictive effects while negative environments producing adverse outcomes (Xu et al., 2024; Yu et al., 2025). In particular, parental styles and parent–child interactions, as key familial factors with strong cumulative temporal effects (McWayne et al., 2004; Knappe et al., 2009), exert direct and long-lasting influences on children’s social–emotional competence development. Given the rapid growth of neural networks and high environmental sensitivity during early childhood (Suzuki, 2010), children’s social and emotional development is more susceptible to being shaped by parenting styles and the quality of interactions. Therefore, it is essential to examine the overall development of young children’s social–emotional competencies and their relationship with these two critical family factors.

Ecosystem theory posits that child development is influenced by both microsystems and exosystems (Rosa and Tudge, 2013). The family serves as the primary and most frequent setting for young children to form interpersonal connections, making familial factors irreplaceable in laying the foundational groundwork for the development of children’s social–emotional competencies (Bronfenbrenner and Morris, 2006; Hooper et al., 2022). Parenting styles serve as the core element continuously operating within this system; symbolic interactionism posits that interpersonal interactions are the key channels for transmitting emotions, norms, and social cognition (Blumer, 1969). The quality of parent–child interactions profoundly shapes young children’s understanding of social relationships and their modes of emotional expression through daily emotional exchanges and behavioral feedback. Parenting styles and parent–child interactions, as critical proximal family processes, operate through fundamentally dynamic and mutually constitutive mechanisms. While their importance for early childhood development is well-established, existing research often assumes simplistic linear relationships when examining their impact on children’s social–emotional competencies. However, certain common phenomena in the reality of child-rearing cannot be fully explained by linear models: among parents who employ strict discipline, some children exhibit good self-control, while others may become socially withdrawn; among parents who adopt a hands-off approach, some children grow up to be willful and impulsive, while others display confidence and independence. These differences suggest that parenting behaviors may not influence children’s development uniformly; rather, they are likely to produce varying degrees of impact depending on the specific context. As research on these two family factors and child development has deepened in recent years, a minority of scholars have begun to use nonlinear analytical methods to explore early childhood development, discovering threshold effects between parenting styles, parent–child interactions, and children’s physical and emotional development (Nicholson et al., 2012; Padilla and Ryan, 2019). Generalized Additive Models (GAM), as effective tools for analyzing nonlinear relationships, can flexibly identify complex patterns of relationships and key inflection points among variables. They have already been preliminarily applied in research on the relationship between teacher-child interactions and child development (Burchinal et al., 2010; Hatfield et al., 2016). Nevertheless, its application in the field of parenting remains quite limited. This implies that exploring the threshold effects of parenting styles on early childhood development within family contexts represents both an extension of nonlinear analytical methods into new domains and a valuable attempt to address the needs of parenting practices. Meanwhile, given that early childhood development progresses rapidly and often exhibits sudden shifts and discontinuities, GAM is better suited than traditional linear methods to capture the potential differential effects of parenting behaviors across different levels. It can effectively identify the “effective range” within which parenting styles have a positive impact and the “risk threshold” beyond which they may have negative consequences. Based on this, this study applies GAM to the context of family upbringing, focusing on examining the nonlinear threshold effects of parenting styles and the quality of parent–child interactions on preschool children’s social–emotional competencies. Building upon existing linear studies, the study aims to provide a new analytical perspective and preliminary empirical insights for understanding the complex effects of parenting behaviors.

## 2 Literature review and hypothesis generation

### 2.1 Effects of parental parenting style and parent–child interaction on children’s social–emotional competence

Parenting styles encompass relatively stable patterns of parental beliefs, behaviors, and emotional attitudes toward their children. Different parenting styles may lead to distinctly different coping behaviors (Bhattacharyya and Pradhan, 2015), emotional experiences, and inner feelings, effectively reflecting the essence of family education and parent–child interactions (Everett, 2011; Li and Xie, 2017), exerting foundational and enduring influences on children’s social–emotional competencies (Garcia et al., 2020; Ren and Pope Edwards, 2015; Zarra-Nezhad et al., 2022). A review of the existing literature reveals that parenting styles can be categorized across multiple theoretical dimensions. Among these frameworks, the model proposed by Baumrind (1967)—which distinguishes between authoritative, authoritarian, and permissive parenting styles—has been widely adopted and has exerted a profound influence within the academic community. Authoritarian parents typically exhibit low levels of responsiveness and high levels of control, frequently resorting to strict disciplinary approaches. This style often leads to parent–child conflicts and significantly increases the risk of children developing issues such as social withdrawal, anxiety and depression, and difficulties with emotional regulation (Fuentes et al., 2015). It exerts substantial negative effects on multiple dimensions of children’s social–emotional development (Speidel et al., 2020) and increases the likelihood of externalizing behaviors like aggression (Ma et al., 2020; Stratton et al., 2014). In contrast, authoritative parents establish explicit behavioral expectations while concomitantly underscoring rational, affectionate, and accepting interactions with children. Empirical evidence has repeatedly demonstrated that this parenting style effectively fosters children’s autonomous exploration abilities and social interaction skills, exhibiting a stable correlation with positive behavioral development (Obaid et al., 2021). Children who are raised in this parenting environment demonstrate optimal personality attributes, including independence, cooperation, friendliness, and sociability. They often demonstrate strong competence and emotional regulation when confronting complex tasks (Nishikawa et al., 2010). Multiple studies indicate that when parents exhibit positive parenting behaviors such as sensitivity and responsiveness, it supports children’s social–emotional competencies (Atzil et al., 2018; Farrell et al., 2019; Morawska et al., 2019). Parents’ positive emotionally instructive parenting styles are effective in predicting fewer externalizing problem behaviors in children later in life (Gottman et al., 1996) and are strongly associated with lower internalizing behavior problems (Rodas et al., 2017; Rodas et al., 2016). In addition, permissive parents, who are emotionally warm but less disciplined and demanding of their children’s behavior, may still have adverse effects of their parenting styles across children’s developmental stages, particularly in self-regulation and rule awareness development (Hosokawa and Katsura, 2019). If parents fail to communicate necessary social rules or family norms to children and consistently encourage the expression of negative emotions, it may lead to a lack of social restraint in such children, potentially increasing the occurrence of rule-breaking and aggressive behaviors (Bayer et al., 2006). According to research on young children, when parents adopt a permissive parenting style, their children generally exhibit difficulty empathizing in social interactions and tend to display a heightened degree of self-centeredness. These characteristics often result in diminished popularity among their peers (Zhao et al., 2018). A review of the literature reveals that differing parenting styles have been shown to yield varying effects on the development of preschool children’s social–emotional competencies. Therefore, the present study posits Hypothesis 1.

*H1(a)*: Authoritative parenting styles positively influence preschool children’s social–emotional competencies.

*H1(b)*: Authoritarian and permissive parenting styles have a negative impact on preschool children’s social–emotional competence.

Beyond the influence of children’s inherent traits, the development of their social–emotional competence is also dependent on the environmental factors in which they live. According to Symbolic Interaction Theory, individuals actively construct their environment, and interpersonal interactions and communication serve as vital means for transmitting diverse information. People engage in social interactions by creating, using, and recognizing symbols attributed to others, thereby achieving self-awareness, understanding situational contexts, and formulating responses (Blumer, 1969). The capacity of children to discern and adequately react to emotional cues during social interactions has been demonstrated to exert a profound influence on the development of their interpersonal relationships (Facci et al., 2024). Among these interactions, parent–child exchanges constitute one of the most prevalent and recurrent patterns children encounter in their daily lives. Children’s ability to recognize and respond appropriately to emotional signals in social interactions will profoundly affect the formation of their interpersonal relationships (Facci et al., 2024), with parent–child interactions being one of the most common and frequent modes of interaction in children’s lives. Parent–child interaction refers to the familial, long-term emotional communication between parents and children (Cao et al., 2023), and the quality of parent–child interaction characterizes the degree of strengths and weaknesses of their interaction behaviors. Early parent–child interactions provide children with initial social interaction scenarios, and parental touch and stimulation have been shown to rapidly activate the brains of infants and foster the development of self-awareness during this process (Wertlieb, 2019). Numerous studies have pointed out that mothers with high levels of sensitivity and interactivity enable young children to maintain superior emotional regulation in other environments outside the home as well (Pratt et al., 2019; Yirmiya et al., 2018). Parental use of interactive approaches such as joint attention and positive affect (vocal and physical) with infants often positively predicts children’s ability to employ appropriate emotional regulation strategies (Abraham et al., 2016). Positive parenting practices and the establishment of a secure parent–child attachment enhance children’s social competencies (Grazuleviciene et al., 2017a). Through the active observation and imitation of parent–child interactions, young children engage with others, thereby developing positive interpersonal skills. Evidently, parent–child interaction exerts positive predictive effects across multiple dimensions of young children’s social–emotional competence, including self-awareness, emotional regulation and control, social competence, and interpersonal skills. Therefore, to investigate its impact on overall social–emotional development, Hypothesis 2 is proposed.

*H2*: The quality of parent–child interactions positively affects the development of preschool children’s social–emotional competencies.

### 2.2 The mediating role of parent–child interaction quality

Parenting styles reflect the cultural logic underlying parental childrearing within the family, while parent–child interactions reveal the specific behaviors and outcomes of these parenting approaches. Existing research indicates that when parents prioritize fostering children’s autonomy, independence, responsibility, and tend to adopt a collaborative, highly responsive authoritative parenting style, it becomes more conducive to establishing warm, respectful, and emotionally close interactions between parents and children (Altintas, 2016; Wilcox, 1998). High-quality parent–child attachment and interaction enhance young children’s social competence and interpersonal interactions (Grazuleviciene et al., 2017b). Conversely, when parents experience elevated levels of stress, their parenting behaviors tend to favor threatening and controlling strategies, which are characterized by low rational control and high power intervention. This can lead to parent–child emotional detachment and reduced quality of interactions (Dodge, 1993). In cross-cultural contexts, a hybrid parenting style combining control and high responsiveness is commonly observed among Chinese mothers: while emphasizing rule compliance and adult authority, they also maintain high sensitivity and support for their children’s needs, creating a more positive emotional atmosphere in parent–child interactions than purely authoritarian parenting (Chao, 1994). To a certain extent, this confirms that parenting styles have a direct impact on the quality of parent–child interaction.

Both parental styles and parent–child interactions are micro-system variables influencing child development. The former is influenced by educational background, economic income, and cultural values (Bronfenbrenner, 1986; Conger and Donnellan, 2007; Xu et al., 2005), representing typical innate factors that are also relatively implicit. Moreover, the quality of parent–child interaction is considered a crucial observable indicator of family parenting standards and significantly predicts young children’s social–emotional development (Junttila et al., 2007). This quality directly reflects diverse parenting styles and is susceptible to variation influenced by other factors (Zhang et al., 2021). Consequently, it frequently functions as an intermediary variable bridging the gap between unchangeable family factors and child development. Symbolic interactionism also emphasizes that individuals’ understanding of themselves, others, and social norms arises from and is continuously shaped through interpersonal interactions (Blumer, 1969), a phenomenon that is particularly evident in parent–child relationships. Young children’s understanding of social norms and acquisition of emotional expression are not the result of passively accepting their parents’ parenting beliefs, but are gradually constructed through each specific parent–child interaction. The way parents respond to their young children’s emotions, their approach to resolving conflicts, and the tone they use when setting behavioral expectations all serve as cues in their interactions that children continually interpret and internalize, ultimately becoming an integral part of their social–emotional skills (LaRossa and Reitzes, 1993). In this process, the quality of parent–child interaction plays an irreplaceable role: parenting styles are transformed from stable emotional tendencies within the family into concrete social experiences that young children can perceive, interpret, and internalize, with parent–child interaction serving as the direct pathway for this transformation to occur. In other words, the quality of parent–child interaction is the ultimate manifestation of parenting styles at the micro-behavioral level, and it also represents the most frequent and consistent form of immediate socialization that young children experience within the family. Other family process variables—including parent–child attachment, which provides an emotionally secure foundation for interaction; parental efficacy, which reflects the subjective aspects of parenting behavior; and the family emotional atmosphere, which sets the overall emotional tone of interaction—are all closely related to parent–child interaction. However, none of these variables can directly replace parent–child interaction as the core channel through which parenting behaviors are transmitted to children’s developmental outcomes. Consistent with this theoretical analysis, previous studies consistently indicate that parents who favor supportive authoritative parenting styles—characterized by encouraging humility and providing protection—tend to prioritize emotional support for young children. This manifests as rational communication on relevant matters, emphasizing persuasion through reason, fostering close parent–child bonds, and positively predicting children’s social and emotional skill development (Scherer et al., 2019; Poulain et al., 2019; Rocha et al., 2020). In contrast, negative parenting styles such as humiliation, overly lax or strict discipline, can alienate the parent–child relationship and decrease the frequency and quality of interactions. This, in turn, may precipitate the manifestation of heightened anger and defiance in young children. Such consequences have the capacity to impede the development of interpersonal skills and emotional regulation (Chen et al., 2000; Nelson et al., 2006). It is worth noting that the empirical evidence cited above primarily comes from cross-sectional studies. Recent longitudinal studies have also found that early authoritative parenting indirectly influences children’s subsequent social skills through the closeness of the parent–child relationship, indicating that parent–child interaction plays a longitudinal mediating role between parenting style and children’s social development (Huang et al., 2024). Zhang et al. (2025) also confirmed in a longitudinal study of Chinese preschool children aged 3–5 that the positive effects of authoritative parenting on young children’s social skills are primarily realized through parent–child interactions. These findings also provide preliminary longitudinal evidence for the present study. Consequently, Hypothesis 3 is hereby proposed.

*H3*: Parental interaction quality mediates the relationship between parenting styles and preschool children’s social–emotional competencies.

### 2.3 Threshold effect of family factors on preschool children’s social–emotional competence

In recent years, much previous research has revealed a linear relationship between factors such as school, family, society and young children’s social–emotional competencies. However, a growing number of researchers have initiated investigations into the existence of critical thresholds in early childhood development with respect to these factors. Notably, numerous researchers have indicated that when teacher-child interaction quality metrics exceed or fall below specific thresholds, their relationship with children’s social and academic outcomes undergoes significant changes (Burchinal et al., 2014; Finch et al., 2015; Leyva et al., 2015; Weiland et al., 2013). A study that examined the quality of teacher-child interactions and their developmental outcomes among preschoolers from low-income families also revealed that when teachers’ emotional support scores exceeded the 5-point threshold, these interactions predicted higher levels of social skills and lower levels of behavioral problems in young children (Burchinal et al., 2010). Other studies have also indicated that when teachers’ emotional support levels reach 6 points or higher, children demonstrate greater emotional self-regulation abilities (Hatfield et al., 2016). Nevertheless, the extant literature on potential critical thresholds or upper limits pertaining to the relationship between family factors and the development of young children’s social–emotional competencies remains limited. Nonlinear analytical approaches are better suited to revealing the variability and complexity of relationships between variables, and they can also provide a more detailed, phased explanation of the patterns of change in these relationships. Based on this, the present study attempts to employ a generalized additive model (GAM) to verify whether parental parenting styles and parent–child interaction quality influence the development of preschool children’s social–emotional competencies. It further explores the nonlinear relationship and underlying mechanisms between these factors, aiming to provide more concrete guidance for the education of preschool children’s social–emotional competencies. Therefore, the study proposes research hypothesis 4.

*H4*: Parenting styles, parent–child interaction quality and preschool children’s social–emotional development exhibit a nonlinear relationship with threshold effects.

## 3 Methods

### 3.1 Participants and procedure

This study randomly selected 7 kindergartens registered in three cities of Guangxi Province, China. From these selected kindergartens, 1–3 classes were randomly chosen from kindergarten junior, middle, and senior classes, totaling 17 classes whose all children were invited to participate in the study. The study surveyed 546 children and their parents; after excluding invalid questionnaires, 504 valid questionnaires were collected, yielding a response rate of 92.31%. All parents of participating children signed written informed consent forms.

The specific procedure consists of two parts. In the first part, researchers conduct one-on-one tests with young children. Prior to administration, the examiner explains the study requirements to the children, then assists them in understanding the questions through oral questioning. The child completed the test by independently selecting specific color boxes (with colors ranging from light to dark representing responses from “does not fit” to “fully fits”). Each child’s testing session lasted approximately 30 min, with breaks permitted if fatigue arose during the process. The second part involved distributing the Parental Authority Questionnaire and Parent–Child Interaction Scale to the children’s parents. Before distributing the questionnaires and evaluating the children’s performance, researchers explained the study’s purpose to kindergarten principals and classroom teachers. After obtaining consent from the kindergarten, an online questionnaire was distributed to parents online via wjx.cn. Parent volunteers were directed to click the provided link to complete an informed consent form and the formal questionnaire. The questionnaire required parents to report their own parenting styles and parent–child interactions.

The study surveyed 546 preschool children and their parents, excluding more than half of the invalid questionnaires that were not answered, obviously disorganized and regularly answered 42 questionnaires, the final valid questionnaires were recovered 504, with a recovery rate of 92.31%. Among them, 143 children were 3 years old (28.38%), 87 were 4 years old (17.26%), 213 were 5 years old (42.26%), and 61 were 6 years old (12.10%); 288 were boys (56.55%) and 216 were girls (43.45%). Among the parents of the children tested: 48 (9.52%) held master’s degrees or higher; 297 (58.92%) held bachelor’s degrees; 102 (20.24%) held high school diplomas; and 57 (11.31%) held vocational school diplomas or lower.

### 3.2 Measures

#### 3.2.1 Measurement of social–emotional competence

This study adopted the Social Skills Improvement System Social Emotional Learning Edition Rating Forms (SSIS SEL) formulated by Gresham et al. (2018), to assess children’s social–emotional competencies. The scale comprises 46 items across five dimensions: self-awareness, social awareness, self-management, interpersonal skills, and responsible decision-making. In order to make the scale more relevant to the cognitive characteristics of children aged 3–6 years, this study took into account the suggestions of frontline kindergarten teachers, and contextualized the linguistic expressions of the scale’s questions without changing the original meaning of the questions as much as possible, so as to make the questions more relevant to the children’s life experiences. During the administration of the test, researchers assisted the children on a one-to-one basis, helping them to understand the meaning of the questions through verbal explanations and observing the children’s responses during the answering process. If the children showed obvious confusion or hesitation about some of the questions, researchers would repeat the questions and situations to facilitate the children’s understanding. Researchers will repeat the question and situation again to facilitate children’s understanding. Researchers will also combine the daily observation of the teacher in charge of the class to assist in judging the reasonableness of the children’s report. The original four-point scoring system (0 = “Disagree,” 3 = “Strongly Agree”) was replaced with a five-point scoring system ranging from 1 to 5 (1 = “Strongly Disagree,” 5 = “Strongly Agree”). The addition of an intermediate option helps to reduce extreme response bias, thus providing a truer picture of the nuances of young children’s social–emotional competence. In the field of assessment targeting young children’s social–emotional competence, the five-point scale has become the commonly used scale format (Ölçer, 2017; López-Cassà et al., 2022). Higher scores indicate that children’s self-perceived strength in the respective skill. In this study, the Cronbach’s Alpha of the form was 0.868. It demonstrated good construct validity: *χ*^2^/df = 2.249, CFI = 0.925, TLI = 0.900, RMSEA = 0.087, GFI = 0.967.

#### 3.2.2 Measurement of parental parenting style

This study adopted the Parental Authority Questionnaire (PAQ) formulated by Buri (1991) to measure parenting styles among parents of preschool children. The scale comprises three dimensions: authoritative, authoritarian, and permissive. The original scale consisted of 30 items. Prior to the assessment, exploratory factor analysis was conducted on the items within each dimension. Items with a factor loading below 0.40 on a single factor, or those exhibiting cross-loadings on two or more factors with a load difference of less than 0.20, were removed. According to this standard, a total of 25 items were ultimately retained, including 11 items for the authoritarian dimension, 10 for the authoritarian-authoritative dimension, and 4 for the permissive dimension. The loadings of the revised items on their respective factors range from 0.412 to 0.852 for the Authoritative dimension, 0.480–0.822 for the Authoritarian dimension, and 0.606–0.727 for the Permissive dimension. The three-factor model accounts for 63.75% of the total variance. Validatory factor analysis confirms that the revised three-factor structure exhibits acceptable model fit. The questionnaire was administered to parents of young children using a 5-point Likert scale (1 = “Strongly Disagree,” 5 = “Strongly Agree”). Higher scores indicate greater frequency of that parenting style. In this study, the Cronbach’s Alpha of the form was 0.837. It demonstrated good construct validity: *χ*^2^/df = 2.405, CFI = 0.917, TLI = 0.868, RMSEA = 0.092, GFI = 0.942.

#### 3.2.3 Measurement of parent–child interaction quality

This study adopted the Brigance Parent–Child Interactions Scale (BPCIS) formulated by Glascoe (2002). The scale comprises 17 items across five dimensions: guidance, closeness, responsiveness, interactivity, and parental efficacy. It employs a 5-point Likert scale (1 = “Rarely,” 5 = “Always”), with items 7, 8, 13, and 16 reverse-scored. Higher scores indicate better parent–child interaction quality. In this study, the Cronbach’s Alpha of the form was 0.826. The scale demonstrated good construct validity: *χ*^2^/df = 2.411, CFI = 0.975, TLI = 0.836, RMSEA = 0.092, GFI = 0.974.

### 3.3 Data analysis

The analytical process in this study is divided into two main parts, with the aim of first identifying the basic structural relationships among the variables and then exploring the specific forms of these relationships. The analysis was primarily conducted using SPSS 25.0 and R 4.1.1. The first phase focused on examining the structural relationships: using SPSS 25 to conduct tests for common method bias, perform descriptive statistics, tests of differences, and correlation analyses on each variable, and employing the SPSS PROCESS macro to conduct mediation analysis. This was done to examine the structural relationships among parenting styles, the quality of parent–child interaction, and young children’s social–emotional competence, to confirm whether the proposed pathways between variables hold true, and to determine whether the quality of parent–child interaction plays a mediating role. Building on the findings from the mediation analysis, which confirmed a significant association among the three variables, the second phase further explored the nonlinear characteristics of this relationship: using Generalized Additive Modeling (GAM) in R to examine the complex effects of two key family factors on the development of young children’s social–emotional competence. The GAM analysis was implemented using the mgcv package, employing thin-plate regression splines as the smoothing basis function for parenting styles, and automatically selecting the optimal smoothing parameters via generalized cross-validation (GCV). Three types of models were constructed for comparison: Model 1 included the quality of parent–child interaction as a linear covariate and parenting style as a smoothed term; Model 2 included only the smoothed term for parenting style; and Model 3 was a standard linear regression model. Model 1 was selected as the primary analytical model because the quality of parent–child interaction serves as a crucial proximal behavioral channel through which parenting styles exert their effects; statistically controlling for this effect allows for a more accurate identification of the independent nonlinear characteristics inherent to the parenting styles themselves. Model selection was based on AIC values and ANOVA tests, with the model yielding the lowest AIC value deemed the best fit. After identifying the inflection point using GAM, segmented linear regression is further employed to estimate the slope coefficients on either side of the inflection point. The location of the inflection point is confirmed by visually inspecting the fitted curve and conducting a log-likelihood ratio test.

## 4 Results

### 4.1 Common method bias test

Since the data in this study employed three separate scales to test preschool children and their parents, a common method bias test was conducted using Harman’s single-factor test to detect potential common method bias. Results showed that the unrotated first factor explained only 20.64% of the total variances, falling below the 40% of the total variances. In conclusion, the data in this study did not exhibit common method bias.

### 4.2 Descriptive statistics and correlation analysis of core variables

Descriptive statistics and correlation analyses were conducted on children’s social–emotional competencies, parental parenting styles, and parent–child interaction quality. Results revealed that preschoolers’ social–emotional competencies (*M* = 3.572 > 3, SD = 0.656) and parent–child interaction scores (*M* = 3.629 > 3, SD = 0.487) both fell within the upper-middle range. Among parenting styles, the authoritative dimension (*M* = 3.656 > 3, SD = 0.418) was moderately high, while the authoritarian dimension (*M =* 2.897 < 3, SD = 0.488) and permissive parenting (*M* = 2.346 < 3, SD = 0.491) were at a lower-middle level. Categorizing parents by their highest mean score across the three dimensions revealed the largest group in the authoritative category (*n* = 363), followed by authoritarian (*n* = 78), and the smallest group in permissive parenting (*n* = 63). Differential tests of other demographic factors revealed that parents were more likely to use authoritative parenting with girls (*t* = 2.479, *p* < 0.05) and permissive parenting with boys (*t* = 2.619, *p* < 0.05). Compared to parents with lower educational attainment, those holding master’s degrees or higher (*F* = 2.190, *p* < 0.05) and bachelor’s degrees (*F* = 2.835, *p* < 0.05) were more likely to adopt authoritative parenting styles, while parents with vocational high school education or below (*F* = 2.736, *p* < 0.05) were more likely to use permissive parenting styles.

Additionally, this study found that parental parenting styles and the quality of parent–child interactions are mutually correlated with children’s social–emotional competencies, as shown in Table 1. Specifically, authoritative parenting styles showed significant positive correlations with both parent–child interaction quality and all measures of social–emotional competence (*r* values ranging from 0.482 to 0.593, *ps* < 0.01). Permissive parenting styles exhibited significant negative correlations with both variables (*r* values ranging from −0.450 to −0.420, *p*s < 0.01). authoritarian parenting showed negative correlations with both variables but these were not statistically significant. Therefore, after controlling for other family factors and excluding authoritarian parenting, mediation analyses were conducted examining the effects of authoritative and permissive parenting styles, parent–child interaction quality, and children’s social–emotional competence.

**Table 1:** Descriptive statistics and correlation analysis results for each variable.

	M	SD	1	2	3	4	5	6
1. Authoritative parenting style	3.656	0.718	1					
2. Authoritarian parenting style	2.897	0.488	0.082	1				
3. Permissive parenting style	2.346	0.696	−0.391^**^	−0.065	1			
4. Parental child-rearing practices	3.143	0.461	0.800^**^	−0.393^**^	−0.690^**^	1		
5. Parent–child interaction	3.629	0.487	0.482^**^	−0.119	−0.450^**^	0.378^**^	1	
6. Children’s social–emotional competence	3.572	0.656	0.593^**^	−0.122	−0.420^**^	0.378^**^	0.462^**^	1

M, Mean; SD, Standard deviation. *p < 0.05, **p < 0.01, ***p < 0.001.

### 4.3 Mediation effect test

The correlation results indicate significant correlations among all variables, meeting the basic criteria for further testing the mediating effect. The mediating effect was examined using Model 4 in the SPSS macro PROCESS developed by Hayes (2022). After controlling for demographic variables such as the children’s age, gender, and parents’ educational attainment, hierarchical regression analysis was employed to test whether authoritative and permissive parenting styles impact preschoolers’ social–emotional competencies through the mediating effect of parent–child interaction quality. The results revealed that authoritative parenting significantly and positively predicted social–emotional competence (*β* = 1.868, *p* < 0.001). Authoritative parenting also exerted a significant direct effect on parent–child interaction quality (*β* = 0.505, *p* < 0.001). Parental-child interaction quality further exerted a significant positive predictive effect on social–emotional competence (*β* = 0.812, *p* < 0.001). Conversely, permissive parenting showed a significant negative predictive effect on social–emotional competence (*β* = −2.857, *p* < 0.001); Permissive parenting also exerted a significant direct effect on parent–child interaction quality (*β* = −1.341, *p* < 0.001), with parent–child interaction quality in turn exerting a significant positive predictive effect on social–emotional competence (*β* = 1.237, *p* < 0.001).

To validate the mediating role of parent–child interaction quality between authoritative parenting, permissive parenting, and social–emotional competence, the Bootstrap method was used to perform 5,000 repeated samples and calculate 95% confidence intervals. The results shown in Table 2 indicate that the mediation effect value for the pathway “authoritative parenting → parent-child interaction → children’s social-emotional competence” is 0.411, with a 95% CI of (0.055, 0.737); The mediation effect value for “permissive parenting style → parent-child interaction quality → young children’s social-emotional competence” was −0.166, with a 95% CI of (−0.233, −0.073). The total effects for the two models were 2.279 and −4.516, respectively, while the direct effects between independent and dependent variables were 1.869 and −2.856, respectively. The Bootstrap confidence intervals for both did not include zero, confirming the existence of these mediating paths. The mediating effects accounted for 18.03 and 36.76% of the total effects, respectively. Parental interaction quality partially mediates the effects of authoritative and permissive parenting styles on preschoolers’ social–emotional competence.

**Table 2:** Bootstrap analysis of mediating effects for authoritative and permissive parenting styles.

		Point estimate	Boot S.E.	95% CI		Proportion of total effect
				Lower	Upper	
Authoritative parenting style	Total effect	2.279	0.237	1.809	2.748	100%
	Authoritative parenting style → Social–emotional competence	1.869	0.264	1.347	2.389	81.97%
	Authoritative Parenting style → Parental interaction quality → Children’s social–emotional competence	0.411	0.169	0.055	0.737	18.03%
Permissive parenting style	Total effect	−4.516	0.764	−6.025	−3.007	100%
	Permissive parenting style → Children’s social–emotional competence	−2.856	0.809	−4.455	−1.258	63.24%
	Permissive parenting style → Parental interaction quality → Children’s social–emotional competence	−0.166	0.041	−0.233	−0.073	36.76%

### 4.4 Threshold analysis

Generalized Additive Models (GAM) are commonly employed to analyze complex nonlinear relationships within data, particularly when threshold effects may exist between variables. Therefore, this study employed GAM to examine whether there is a nonlinear relationship between key family factors and young children’s social–emotional competence. Authoritarian and permissive parenting styles were used as independent variables, and after controlling for children’s age, gender, parents’ educational attainment, and the quality of parent–child interaction, the five subscales of young children’s social–emotional competence were analyzed as dependent variables in Table 3. According to the results, the EDF indicator is no <1. It can be seen that, except for the authoritative parenting style showing a linear relationship with children’s self-management abilities, all other factors exhibit non-linear relationships.

**Table 3:** Generalized additive model results for parenting styles and children’s social–emotional competence.

	Self-awareness			Self-management			Social cognition			Interpersonal skills			Responsible decision-making		
	edf	*F*	△*R*^2^	edf	*F*	△*R*^2^	edf	*F*	△*R*^2^	edf	*F*	△*R*^2^	edf	*F*	△*R*^2^
Authoritative parenting style	4.074	20.031	0.373^***^	–	–	–	3.741	11.323	0.234^***^	3.973	19.735	0.365^***^	3.776	12.801	0.262^***^
Permissive parenting style	5.122	7.705	0.214^***^	3.917	5.397	0.127^***^	3.487	12.842	0.251^***^	4.486	8.7867	0.221^***^	5.578	6.628	0.203^***^

*p < 0.05, **p < 0.01, ***p < 0.001.

Based on this, models 1 and 2 representing the two parenting styles are constructed as nonlinear models. Model 1 is a generalized additive model with parent–child interaction quality and both parenting styles as independent variables. Model 2 is a generalized additive model with only parenting styles as independent variables. Model 3 is a regression model with linear parent–child interaction quality and both parenting styles as independent variables. Model 1 < − gam (Children’s social–emotional competence ~ parent–child interaction quality + s (parenting style)), Model 2 < − gam (Children’s social–emotional competence ~ s (parenting style)). ANOVA results showed no significant statistical difference between Model 1 and Model 2. Additionally, Model 1 had a lower AIC than Model 2, making it the optimal model and the final selection.

Model 1 testing confirmed that the inflection point for the impact of authoritative parenting on children’s social–emotional competence was 3.9 points (log-likelihood ratio test *p*s < 0.0001), as shown in Table 4. Specifically, when authoritative parenting scores were below 3.9 points, children’s social–emotional competence scores increased rapidly; however, beyond 3.9 points, although scores continued to rise, the rate of increase slowed. For permissive parenting styles, inflection points between 2.5 and 3.7 points (log-likelihood ratio test, *p* < 0.0001). Specifically, when permissive parenting scores ranged between 2.5 and 3.7 points, children’s social–emotional competence scores dropped sharply. Beyond 3.7 points, scores continued to decline overall, with no significant threshold effect observed below 2.5 points.

**Table 4:** Comparison of predictor coefficients (β) across models.

	Children’s social–emotional competence						ANOVA test	
	Model 1		Model 2		Model 3		Model 1 vs. Model 3 (*p’*)	Model 2 vs. Model 3 (*p’*)
	β	SE	β	SE	β	SE		
Parental interaction quality	0.709^**^	0.255	—	—	0.223^***^	0.025	<0.001	<0.001
Authoritative parenting style	—	—	—	—	0.489^***^	0.026	—	—
Score ≤3.9	3.827^**^	0.134	1.784^**^	0.211	—	—		
Score >3.9	2.433	0.158	1.662^**^	0.396	—	—		
AIC	1536.153		1541.025		1544.304			
Adjusted R-squared^2^	0.426		0.406		0.387			
Parental interaction quality	1.083***	0.270	—	—	−0.263^***^	0.027	—	—
Permissive parenting style	—	—	—	—	0.340^***^	0.081	—	—
score ≤2.5	0.211	0.012	0.970*	0.315	—	—		
2.5≥ score >3.7	−3.415^***^	1.283	−1.354^***^	0.376	—	—		
score >3.7	−0.377	0.023	−0.022	0.004	—	—		
AIC	1566.49		1578.972		1576.657			
Adjusted R-squared^2^	0.314		0.259		0.266			

#### 4.4.1 Threshold effects of authoritative parenting style on subcomponents of children’s social–emotional competence

**Figure 1:**
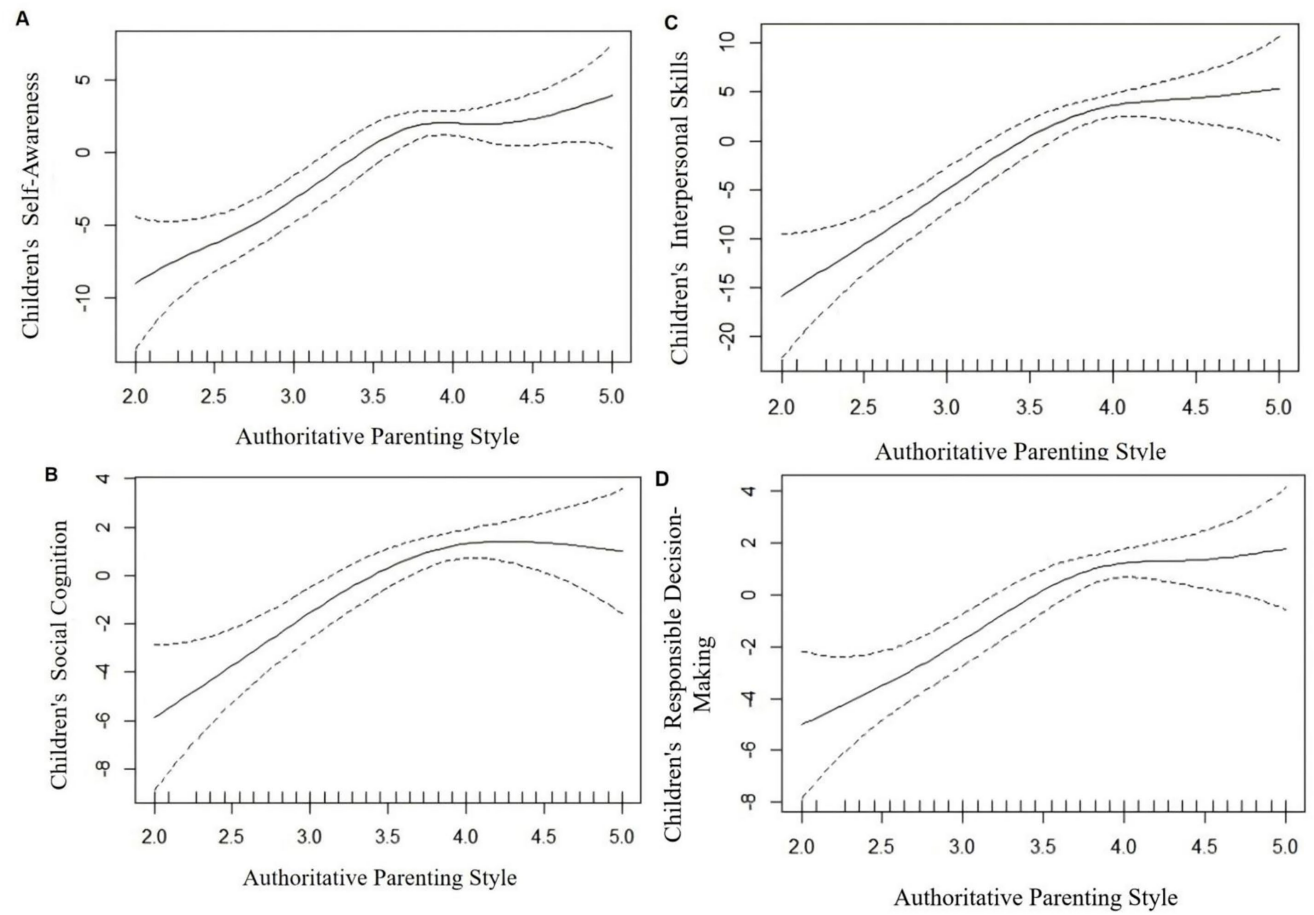
The smooth curve fitting of authoritative parenting style predicting children’s self-awareness **(A)**, social cognition **(B)**, interpersonal skills **(C)**, and responsible decision-making **(D)**, respectively.

As shown in the results of the generalized additive model fit in Figure 1, there is a critical inflection point in the relationship between authoritative parenting and young children’s self-awareness, at approximately 3.8 points (log-likelihood ratio test, *p* < 0.001). The piecewise linear model fitted based on this inflection point indicates that, within the range of scores below 3.8, authoritative parenting is significantly positively associated with self-awareness(*β* = 6.9, *p* < 0.001), and the fitted curve shows a steep upward trend. When scores exceed 3.8, the slope is no longer significant (*p* = 0.183), and the curve flattens, suggesting that the beneficial effect of the parenting style tends to level off. There is a critical inflection point for its relationship with young children’s social cognition, interpersonal skills, and responsible decision-making, which is approximately 3.9 (log-likelihood ratio test, *p*s < 0.001). Analysis using a piecewise linear model revealed that, within the range of scores below 3.9, an authoritative parenting style was significantly and positively associated with all three of the aforementioned competencies (Social Cognition: *β* = 4.4, *p* < 0.001; Interpersonal Skills: *β* = 11.3, *p* < 0.001; Responsible Decision-Making: *β* = 3.9, *p* < 0.001), with the fitted curve showing a steep upward trend. When scores exceeded 3.9, the slopes of all models were no longer significant (*p*s > 0.739), and although the curve continued to rise, the rate of increase flattened.

#### 4.4.2 Threshold effects of permissive parenting style on subcomponents of children’s social–emotional competence

**Figure 2:**
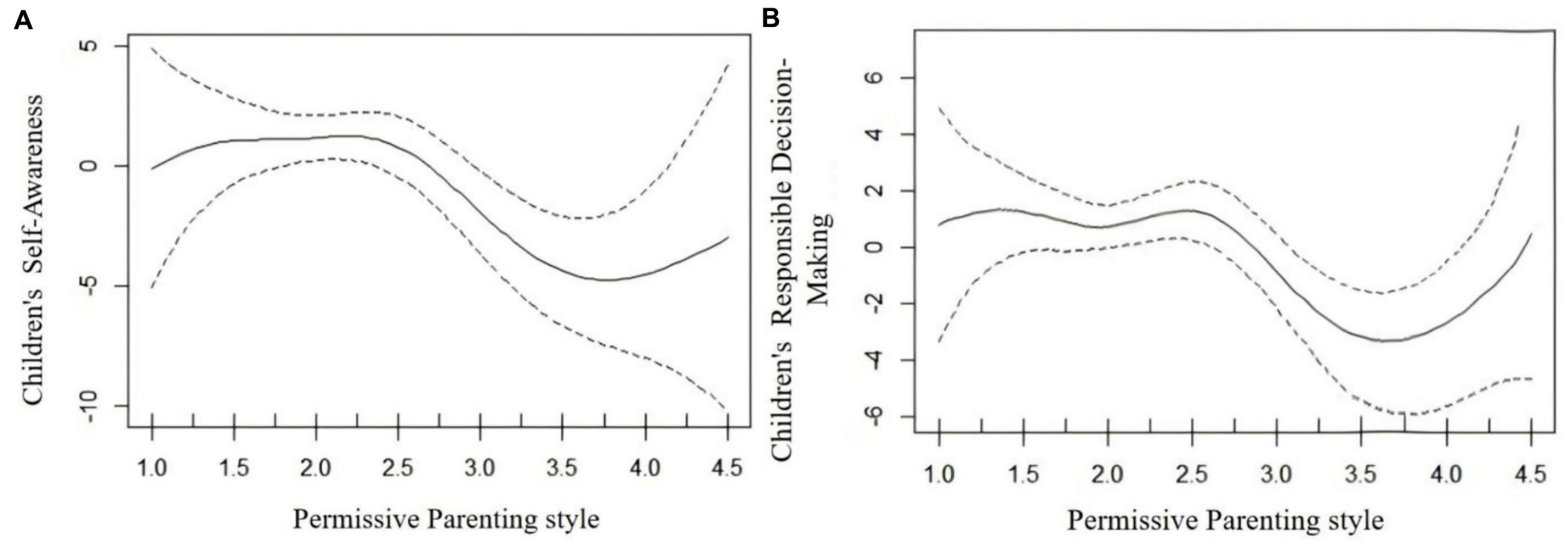
The smooth curve fitting of permissive parenting style, respectively, predicting children’s self-awareness **(A)** and responsible decision-making **(B)**, respectively.

The results shown in Figure 2 indicate that permissive parenting exhibits two critical inflection points for children’s self-awareness and responsible decision-making at scores of 2 and 3.5–3.7, respectively (log-likelihood ratio tests, *p*s < 0.05). Based on this, scores were categorized into three intervals: K1 < 2; 2 ≤ K2 < 3.5; K3 ≥ 3.5. Analysis using a piecewise linear model revealed that, within the range of 2 to 3.5 points, a permissive parenting style was significantly negatively associated with young children’s self-awareness (*β* = −9.0, *p* < 0.001), with the fitted curve showing a steep downward trend; in the ranges below 2 points and above 3.5 points, the slopes were not significant (*p*s > 0.739). The inflection points for children’s responsible decision-making were 2.5 and 3.7 (log-likelihood ratio test, *p*s < 0.05), based on which the data were divided into three intervals: K1 < 2.5; 2.5 ≤ K2 < 3.7; K3 ≥ 3.7. Analysis using a piecewise linear model indicated that, within the range of 2.5 to 3.7 points, a permissive parenting style was significantly negatively associated with children’s decision-making (*β* = −8.2, *p* < 0.001), with the fitted curve showing a steep downward trend; In the intervals below 2.5 and above 3.7, the slopes were not significant (*p*s > 0.770).

**Figure 3:**
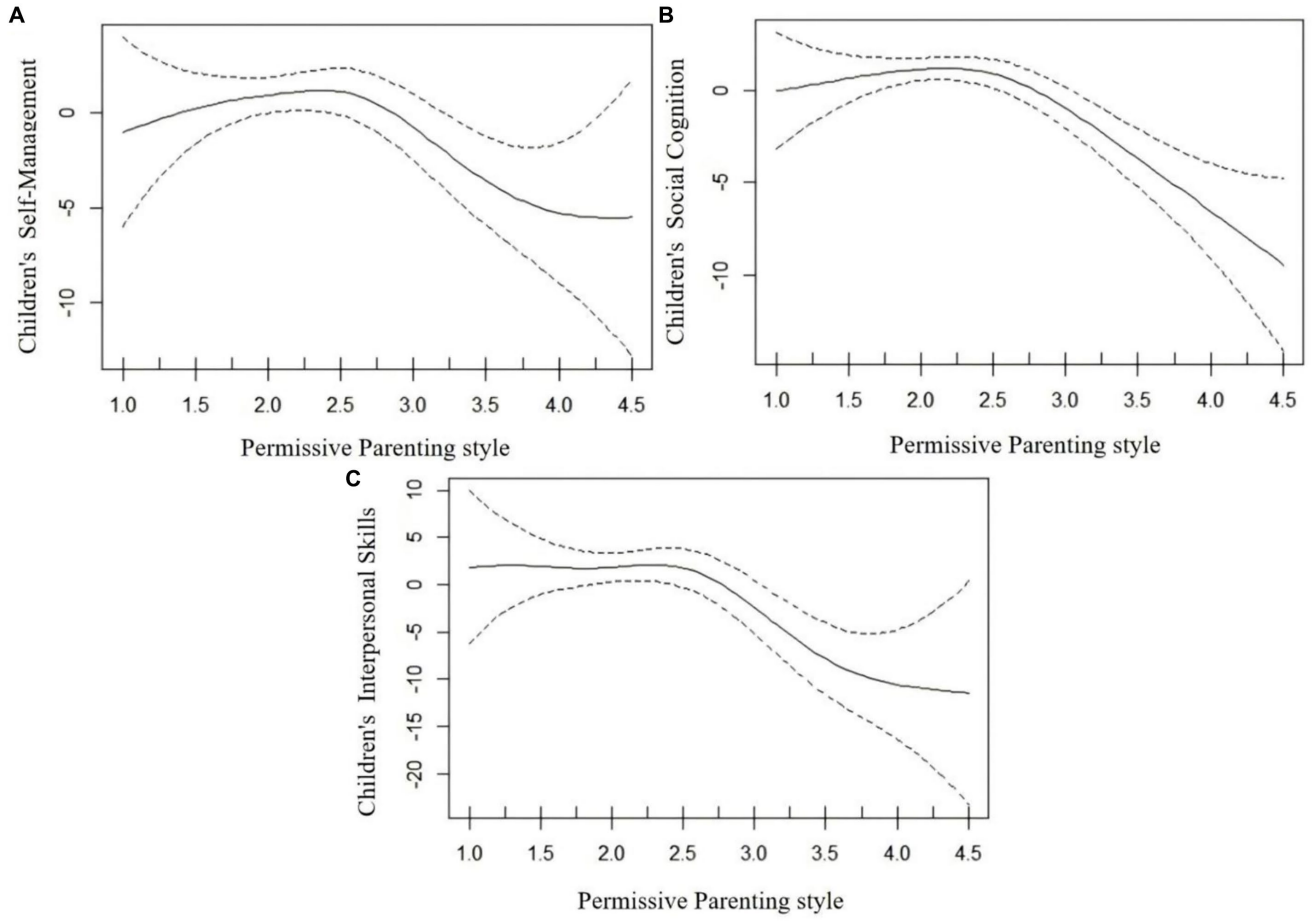
The smooth curve fitting of permissive parenting style, respectively, predicting children’s self-management **(A)**, social cognition **(B)**, and interpersonal skills **(C)**, respectively.

In addition, the results shown in Figure 3 indicate that a single critical inflection point at 2.5 points was identified for the other three domains (log-likelihood ratio test, *p*s < 0.001). Analysis using a segmented linear model revealed that, in the score range above 2.5, permissive parenting was significantly negatively associated with all three of the aforementioned competencies (Social Cognition: *β* = 7.3, *p* < 0.001; Self-Management: *β* = −6.5, *p* < 0.001; Interpersonal Skills: *β* = −12.7, *p* < 0.001), and the fitted curve showed a steep downward trend; in the range below 2.5 points, the slopes of all models were not significant (*p*s > 0.770), and although the curve declined, it tended to flatten out.

## 5 Discussion

### 5.1 Parental parenting styles and parent–child interaction quality as key factors and primary effects in early childhood social–emotional competence development

After statistically controlling for other demographic factors within the family, it was found that parental parenting style and parent–child interaction quality collectively influence children’s social–emotional competence as key family factors, with the former exerting a stronger effect. This aligns with evidence from prior research (Scaramella and Leve, 2004). Ecosystem theory posits that the family constitutes the core microsystem for child development. Parental styles and the quality of parent–child interactions directly form the primary content of this microsystem, while being indirectly influenced by exosystems (e.g., parental work stress, social support networks) and macrosystems (e.g., cultural values, childcare policies). Thus, they serve not only as key proximal processes that can be modified, but also as vital hubs connecting broader ecological contexts to child development outcomes (Bronfenbrenner and Morris, 2006). Parental parenting styles and parent–child interaction quality themselves significantly predict children’s social–emotional competence. Compared to other relatively fixed family factors (e.g., parental education, family structure), these elements are more amenable to adjustment and exert a sustained, flexible influence on the development of young children’s social–emotional competence.

Moreover, parent–child interaction is fundamentally a process of meaning-making and learning through daily exchanges. In the development of their social–emotional competencies, children understand themselves, others, and behavioral norms through sustained interactions with their parents, thereby internalizing social–emotional patterns (LaRossa and Reitzes, 1993). Parenting styles, as attitudes, typically form during early childhood. The beliefs and emotions embedded within these styles are transmitted to children through each specific parent–child interaction, exerting a lasting influence on their early development. Young children complete their entire socialization process by imitating their parents’ patterns of interacting with themselves and others, exerting a lasting influence and primacy effect on the development of their social–emotional competencies (Locati et al., 2025). Simultaneously, parental styles largely determine the patterns and outcomes of parent–child interactions (Lareau, 2003), jointly influencing children’s social–emotional competencies alongside the quality of these interactions. By identifying parenting styles and the quality of parent–child interactions as key factors directly influencing young children’s social–emotional competencies with a priority effect, this study underscores the importance of focusing on and optimizing parental child-rearing practices and the quality of daily parent–child interactions. Future efforts should involve targeted family education guidance and support to help parents enhance their parenting skills and improve interaction quality, thereby effectively promoting the healthy development of children’s early social–emotional competencies.

### 5.2 The direct predictive effects of parental parenting styles on children’s social–emotional competence

This study indicated that authoritative parenting positively predicted children’s social–emotional competencies, while permissive parenting had the opposite effect. And authoritarian parenting showed no significant predictive effect. This stems from authoritative parents typically providing stable, nurturing external environment alongside positive emotional support. Through responsive and warmly supportive behaviors, they foster children’s effortful control, which significantly predicts later effortful control (Karreman et al., 2008). This helps young children develop management of their own emotions and behaviors, thereby comprehensively enhancing their social–emotional competencies. Conversely, under the influence of overly permissive parenting, children tend to become more self-centered in social interactions, leading to poor impulse control during social engagements (Lee et al., 2013) and hindering the development of peer relationships and emotional regulation skills.

Additionally, this study found that authoritarian parenting styles did not significantly predict outcomes, which may be related to differing cultural contexts. Previous research has indicated that in East Asian collectivist cultures, parental control over young children is viewed as a duty to care for them and teach them to avoid risks (Chao, 1994; Wang et al., 2015). Strict parental discipline encourages young children to maintain prosocial behavior in social interactions; children often interpret this as a sign of parental responsibility and care. This, in turn, suppresses certain negative social behaviors and fosters the development of social–emotional skills (Rudy and Grusec, 2006), whereas in cultures that emphasize Western individual autonomy, the same degree of control is more likely to be experienced as a suppression of the self. The excessive control exerted by authoritarian parents can delay young children’s self-awareness development, inhibit peer interactions, and lead to social withdrawal (Neppl et al., 2019), making these negative effects particularly pronounced. Thus, even with lower responsiveness in emotional attentiveness, authoritarian parenting exerts a unique positive effect on East Asian children’s social–emotional development through compensatory and offsetting mechanisms, showing no significant correlation. This further demonstrates that parental parenting style selection and implementation also follow endogenous cultural logic. In addition, the scores for authoritarian parenting in this sample showed little variation, and the partial correlation between authoritarian and authoritative parenting may also have had some influence on the aforementioned non-significant results.

### 5.3 Mediating role of parent–child interaction quality

This study indicated that parent–child interaction quality directly influences children’s social–emotional competencies while also partially mediating the relationship between parenting styles and these competencies. This finding is consistent with previous research (Poulain et al., 2019). The underlying reason is that when parents respond to children’s socialization needs with affection and warmth while providing high-quality companionship, it fosters positive parent–child interactions and inspires children to acquire social skills through observation and imitation (Yu et al., 2025), directly influencing their social–emotional development. Conversely, when parent–child interaction is scarce and responsiveness is low, young children lack both interaction partners and feedback on the effectiveness of their social engagements. This impedes the development of their interpersonal and social cognitive abilities, thereby demonstrating that the quality of parent–child interaction directly and positively predicts children’s social–emotional competence. Furthermore, authoritative parenting involves both reasonable control and respect for children, fostering high-quality parent–child interactions and positively predicting reduced negative emotions in young children (September et al., 2016; Zhang et al., 2025).

Permissive parenting styles may foster weaker parent–child bonds due to neglectful treatment of young children, potentially hindering the development of secure attachment. Prolonged exposure to chronic stress can lead to reduced hippocampal volume in children and alter the structure of the amygdala and prefrontal cortex—areas closely linked to emotional regulation and social behavior—thereby impeding the development of their social–emotional competencies (Lupien et al., 2018). On the other hand, another manifestation of permissive parenting involves excessive indulgence of young children, which can cultivate a spoiled personality in them. This hinders the development of empathy, resulting in weaker interpersonal skills and self-management abilities (Golovey et al., 2016). Enhancing the quality of parent–child interactions can help children understand basic social norms and develop greater empathy (Liu et al., 2022). This can partially mitigate the harmful effects of negative parenting styles, serving as a mediating factor.

### 5.4 Threshold effects of authoritative and permissive parenting styles

Results indicate that when authoritative parenting scores fall below the 3.8–3.9 threshold, children’s self-awareness, social cognition, interpersonal skills, and responsible decision-making abilities show rapid improvement. Beyond this threshold, the rate of improvement slows, demonstrating a threshold effect. On the one hand, authoritative parents typically adopt warm and supportive emotional attitudes, enriching children’s self-awareness and social cognition, enhancing their social adaptability, and promoting development across multiple dimensions of social–emotional competence (Liu et al., 2023). On the other hand, the positive effects of the authoritative parenting style begin to level off at scores above 3.9. This phenomenon can be understood in terms of the law of diminishing marginal returns. The core characteristic of the authoritative parenting style lies in the combination of warm responsiveness and reasonable expectations: parents remain sensitive and accepting of their children’s emotional needs while setting age-appropriate behavioral expectations and providing consistent guidance. As parenting scores rise from low to moderate levels, increased emotional support and improved behavioral guidance from parents have the most direct impact on promoting young children’s social–emotional competencies; however, once a high level is reached, the independent contributions of these two forms of support to children’s development gradually plateau. At this stage, young children’s basic needs for emotional security have been relatively stably met, and reasonable behavioral expectations have been established. Although further optimization of parenting styles remains beneficial, its marginal effects are no longer as significant as they were during the earlier stages of improvement (Baumrind, 1967). Meanwhile, from the perspective of ecosystem theory, once the quality of care within the family microsystem reaches a certain level of adequacy, the further development of young children’s social–emotional competencies becomes increasingly dependent on the synergistic effects of other microsystems, such as peer interactions and teacher-child relationships; consequently, the independent benefits of caregiving approaches tend to diminish marginally. In practical terms, this finding suggests that family education guidance should focus on helping parents adopt and maintain a basic authoritative parenting style. For example, this involves being sensitive to young children’s emotional needs and responding appropriately, holding reasonable expectations for their behavior and providing consistent guidance, while also prioritizing the quality of parent–child interaction as a more practical and sustainable approach to intervention. Cross-cultural comparative studies also support the universality of the positive effects of authoritative parenting. Haslam et al. (2020) compared Australia, a society with an individualistic cultural context, and Indonesia, a society with a collectivist cultural context, and found that authoritative parenting was significantly associated with higher emotional regulation abilities and fewer behavioral problems in young children in both cultures; A meta-analysis by Pinquart and Kauser (2017) also indicated that authoritative parenting was associated with at least one positive aspect of children’s emotional and social development across all regions examined. However, it is worth noting that this paper also pointed out that effect sizes may vary across different cultural regions; therefore, whether the threshold for the diminishing returns observed in this study holds true in non-East Asian cultural contexts remains to be tested in future research. Additionally, this study found that the development of self-management skills among children under authoritative parenting follows a linear trajectory. This outcome stems from the fact that children raised authoritatively scored lowest on self-management abilities, indicating weaker emotional and behavioral regulation. This may stem from the “high demands, high responsiveness” nature of authoritative parenting. Such parents tend to exercise strict oversight over their children’s behavior, fostering dependency to some extent. Consequently, children develop relatively weaker self-discipline and self-control, instead being more consistently and stably influenced by their parents’ authoritative parenting model.

Under permissive parenting, children’s self-management, interpersonal, and social cognitive abilities showed no significant changes in indulgence levels below 2.5 point threshold. A marked decline only emerged after this threshold. This indicates that permissive parenting, characterized by excessive indulgence and lax supervision, can lead to observable issues in children’s social interactions, such as peer interaction difficulties and negative emotions. If not promptly corrected and the permissiveness level rises to the warning threshold, it will exert a profoundly negative impact on multiple aspects of children’s social–emotional development (Shafipour et al., 2015). The fact that permissive parenting styles show a shift from no significant harm to a rapid decline around the 2.5-point mark can be explained from the perspective of proximal processes in ecosystem theory. When parenting style scores fall below 2.5, parents may merely be more lenient in terms of behavioral expectations, yet the basic functions of parent–child interaction—such as emotional responsiveness and daily communication—remain intact. However, once scores cross the 2.5–3.7 range, the “low expectations, low constraints” characteristics of permissive parenting gradually emerge, and the guiding and normative functions of parent–child interaction are significantly weakened. It is worth noting that this study found that the quality of parent–child interaction itself has a consistent positive effect on young children’s social–emotional competence. This implies that when parents are overly permissive, the socializing function of parent–child interaction—a protective factor—is also undermined, leading to concentrated difficulties in areas such as self-regulation, social cognition, and interpersonal relationships (Hosokawa and Katsura, 2019). From a practical perspective, a score of 2.5 can serve as a reference threshold that should raise concerns in the context of family education, indicating that parents should avoid allowing their parenting practices to remain in the realm of excessive permissiveness over the long term, and should prioritize improving the quality of parent–child interaction to mitigate the potential negative effects of such parenting styles. In addition, there may be cultural differences in the impact of permissive parenting styles on young children’s social–emotional competence: Xia et al. (2020) found that permissive parenting styles were associated with lower levels of social–emotional readiness among Chinese preschoolers. However, a recent systematic review noted that in cultures that prioritize relational harmony over behavioral autonomy—such as Spain, Italy, and Brazil—parents who adopt a permissive parenting style tend to exhibit non-controlling, non-punitive, and tolerant behavioral patterns, which are consistently positively associated with better mental health and social adjustment in children (Widiastuti et al., 2026). Therefore, the risk thresholds identified in this study may reflect characteristics specific to East Asian cultural contexts. Future research could build on these findings to further examine whether cultural background moderates the valence and effect size of permissive parenting styles.

Additionally, two critical thresholds exist for self-awareness and responsible decision-making under this parenting style (scoring 2 points and 3.5 points, 3.7 points respectively). These represent threshold effects where both abilities exhibit steep declines only within the critical range as indulgence increases, with no significant differences observed in other score segments. A score below 2 indicates a low-scoring range, suggesting that while parents may exhibit a tendency toward permissive parenting but not significantly. A certain “threshold effect” is present, meaning the behavior has not yet reached the critical threshold that significantly impairs the child’s social–emotional development. However, indulgent and overprotective treatment of young children can induce excessive emotional arousal during interpersonal interactions and emotional regulation, making it harder for them to control negative emotions and behaviors (Kim et al., 2018), thereby substantially amplifying the negative impact on their social–emotional competencies. Moreover, young children’s self-awareness and responsible decision-making abilities are largely developed through interactions with peers. Therefore, when permissive parenting scores reach higher levels (3.5 points or above), reduced parental oversight allows these abilities to be more heavily influenced by other individuals within the child’s social network, resulting in non-significant outcomes.

### 5.5 Research implications and future directions

This study has certain limitations in terms of time and scope, and the following aspects should be considered when interpreting and generalizing its findings. Firstly, the nonlinear relationships and thresholds identified in the cross-sectional design represent correlational findings and do not yet allow for the direct inference of causal relationships; these should be tested in future studies using longitudinal designs. Secondly, since the sample was drawn entirely from a single region in China, caution is warranted when generalizing the identified threshold characteristics to other cultural contexts; future research should expand the sample size and include different cultural groups to facilitate systematic comparisons. Although the study included tests for common-method bias and utilized instruments with established reliability and validity, and although the children’s self-reports were adapted to specific contexts and administered on a one-on-one basis, the stability of self-assessments at this age remains limited. Parents’ self-reports may also be influenced by social expectations; in future studies, these findings could be cross-validated using teacher reports or behavioral observations. In addition, this study did not collect data on family structure, income, or parental age; these factors may moderate the effects of parenting styles. Future research could incorporate them into the analytical framework to further test the robustness of the findings. Furthermore, while this study primarily examined the independent effects of each parenting dimension, the impact of mixed parenting styles remains to be explored in subsequent research.

## 6 Conclusion

This study examined the mechanisms through which parental parenting styles and parent–child interaction quality influence preschool children’s social–emotional competence. Findings confirm that both are key familial factors influencing children’s social–emotional competencies, with parent–child interaction partially mediating the relationship between parenting styles and social–emotional development. Generalized additive model analysis further reveals distinct threshold effects for both authoritative and permissive parenting styles on children’s social–emotional competencies, indicating a significant nonlinear relationship. These findings provide theoretical grounding and practical support for families cultivating preschool children’s social–emotional capabilities. By integrating ecological systems theory and symbolic interactionism, this study uncovers the nonlinear threshold mechanism underlying these influences, offering a fresh perspective on the complex relationship between family environments and child development. In practical terms, these findings suggest that parents may wish to consider the combined effects of parenting styles and the quality of parent–child interactions, and consciously adjust their behavior in accordance with the relevant threshold ranges. Given the cross-sectional design of this study and the characteristics of the regional sample, these recommendations should be viewed as preliminary, exploratory insights; their applicability in broader contexts requires further testing in future research.

## Data Availability

The raw data supporting the conclusions of this article will be made available by the authors, without undue reservation.
